# Application of essential oil of *Pistacia atlantica* Gum, polypropylene and silica nanoparticles as a new milk packaging

**DOI:** 10.1002/fsn3.1660

**Published:** 2020-06-16

**Authors:** Hasan Ellahi, Elham Khalili Sadrabad, Seyedhossein Hekmatimoghaddam, Ali Jebali, Elham Sarmast, Fateme Akrami Mohajeri

**Affiliations:** ^1^ Zoonotic Diseases Research Center Department of Food Hygiene and Safety School of Public Health Shahid Sadoughi University of Medical Sciences Yazd Iran; ^2^ Department of Advanced Medical Sciences and Technologies School of Paramedicine Shahid Sadoughi University of Medical Sciences Yazd Iran; ^3^ Medical Biotechnology Research Center Ashkezar Branch Islamic Azad University Yazd Iran; ^4^ Department of Food Hygiene and Quality Control Faculty of Veterinary Medicine Shahrekord University Shahrekord Iran

**Keywords:** antibacterial activity, milk packaging, *Pistacia atlantica*, silica nanoparticle

## Abstract

The aim of current study was to investigate the antimicrobial effect of gum essential oil of *Pistacia atlantica* (wild pistachio) tree (GEO) and design a new film based on polypropylene polymer coated with silica nanoparticles and GEO. The antimicrobial activity of the packaging film was evaluated with or without milk on *Staphylococcus aureus*, *Salmonella enterica*, *Escherichia coli*, and *Listeria monocytogenes* during 35 days. The results showed that GEO has significant antibacterial properties. It was most effective on *Salmonella enterica*, while its effect on *Listeria monocytogenes* was the weakest. Antimicrobial activity of the film without milk showed no significant differences among the different sizes of nanoparticles used (0.05, 0.025, and 0.051 g) (*p* ≥ .05). It can be concluded that polypropylene incorporated with GEO and silica nanoparticles active film had antimicrobial properties up to 35 days, while using with milk or without milk. Therefore, this type of packaging is effective to enhance the shelf life of milk.

## Graphical abstract

The aim of current study was to investigate the antimicrobial effect of gum essential oil of Pistacia atlantica (wild pistachio) tree and to design a new package containing silica nanoparticles coated with this essential oil on a polypropylene bed.

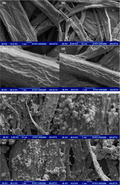

## 1 INTRODUCTION

Packaging, in special word food packaging, is considered a complex, dynamic, scientific, artistic, and controversial part of the business. Consumers demand has also increased for foodstuffs with higher quality, longer shelf life, and also ready‐to‐use foods that remain fresh in natural condition for longer periods. The packaging materials used for food products should provide optimum maintenance properties for the intended duration of use (Rastiani et al., 2019; Taylor et al., 2014). Active packaging is a kind of packaging that improves safety, durability, and sensory properties of the foodstuff, besides having the main properties of common packaging (Rodriguez, Batlle, & Nerin, 2007; Sarmast, Fallah, Dehkordi, & Rafieian‐Kopaei, 2019). Antimicrobial packaging, as an exciting innovation in active packaging, provides the controlled release of antimicrobials from the packaged materials (Akrami Mohajeri, Riahi, Khalili Sadrabad, Hekmatimoghaddam, & Jebali, 2019). Antimicrobial agents in packaging materials can increase the products' shelf life by preventing corruption and bacterial growth (Véronique, 2008).

Antimicrobial film is effective against a wide range of microorganisms at low concentrations while maintain the packed food without any changes in its sensory properties. Moreover, this type of packaging is not expensive and is consistent with the existing rules (Kerry, O’Grady, & Hogan, 2006). Antimicrobial and antioxidant activities are the main goals of researches on active packaging (Akrami et al., 2015). Natural antimicrobials inhibit and retard the growth of microorganisms, hence prevent the spread of various foodborne diseases (Abdoul‐Latif et al., 2010). Studies conducted in this regard have been indicated that using antimicrobial films is much more effective than direct addition of antimicrobials into foodstuff as these active compounds could be released slowly from the packaging to the surface of food and remained in the required concentration to prevent microbial growth (Appendini & Hotchkiss, 2002; Mohajeri et al., 2018; Rahimi et al., 2019; Silveira et al., 2007).

Essential oils (EO_S_) vary in function and composition depending on the type of plant, climatic conditions, and drying and processing methods. Mediterranean countries have a strong background in using EOs as antioxidants and antimicrobials (Akrami et al., 2015; Rahimi et al., 2019). *Pistacia atlantica* tree (*Pistacia atlantica subsp. Kurdica*) is a wild pistachio plant from the *Anacardiaceae* family (Sharifi & Hazell, 2011). *Pistacia atlantica* tree is grown mainly in western and less in the central and eastern part of Iran which has been used by local people as ingredients in some food, jams, and also gum (Hatamnia, Abbaspour, & Darvishzadeh, 2014). The essential oil derived from gum contains α‐thujene, α‐pinene, camphorene, sabinene, β‐pinene, ∆3‐carene limonene, and also the alpha‐trapezoid neol, armandander, and caprylic acid (Sharifi & Hazell, 2011).

The aim of this study was to design a new polypropylene package for milk, which is coated by nano‐scale of GEO, and investigate its antimicrobial properties in vitro and in a food model.

## 2 MATERIALS AND METHODS

### 2.1 Preparation of suspensions of nanoparticles and Essential oil

GEO was obtained from *Pistacia atlantica* tree by Clevenger's apparatus. Silica nanoparticles (Merck, Germany) with approximate size of 100 nm were used for further stabilization of the GEO molecules and prevent its rapid evaporation. Three concentrations (0.05, 0.025, 0.001 g) of silica nanoparticles separately mixed with 500 μl of nondiluted GEO in an acid‐resistant polyethylene container. It was heated and mixed intensely on the shaker for 10 min. The achieved concentrated suspension was used for the following tests.

### 2.2 Preparation of active packaging

For each treatment, a piece of 6 × 6 cm sheet of polypropylene polymer was used and 10 tiny pores of about 100 µm made manually in one side of label by needle before sterilization with 70% ethanol. Then, 4 layers of 3 × 3 cm Whatman cellulose paper were placed between the 2 layers of polypropylene films. Three edges of the films were sealed by heat. Then, each suspension contains specific concentration of GEO—silica nanoparticle was injected into the Whatman paper layer with a sterile syringe. Finally, the free (fourth) edge of the package was sealed by heat (Rastiani et al., 2019).

### 2.3 In vitro antimicrobial active packaging

Antibacterial activity of the package was investigated by vapor diffusion method. The films were placed on inner surface door of Mueller–Hinton agar (Merck) plates which surface inoculated with 10^6^ CFU/ml of microorganisms. The plates were incubated at 37°C for 24 hr. For each of the above samples, a control medium, coated with paraffin, was run, too. Inhibition of microorganisms' growth was studied by counting the number of colonies compare to control group. The experiment was operated in triplicate for 4 samples (Adel, Abedian Amiri, Divband, Safari, & Khalili, 2015; Akrami et al., 2015).

### 2.4 Antimicrobial activity of active packaging in milk

For the antibacterial activity test in milk, *Staphylococcus aureus*, *Salmonella enterica*, *Escherichia coli*, and *Listeria monocytogenes* cultures obtained from the culture collection of Yazd University were used. The cultures of bacterial were grown on nutrient agar slants and kept at 4°C. A portion of 700 μl of the suspension containing 10^6^ CFU/ml each bacterium was inoculated into sterile containers containing 70 ml of commercial low‐fat sterile milk obtained from a local market. In the next step, the prepared package with GEO and 0.001 g silica nanoparticles was put inside the milk container after sterilization with alcohol, and the milk container placed in refrigerator at 4°C. The colonies were counted on days 1, 3, 7, 14, 21, 28, and 35 (Adel et al., 2015).

### 2.5 Scanning electronic microscopy

Surface and cross‐sectional imaging of the packages were performed by scanning electron microscopy (*SEM*) (EM3200 model, KYKY Corporation) at various magnifications. In order to determine the structure of nanoparticles, the 26 KW *SEM* was used. To have cross‐sectional image of the surfaces, a small slice of films was placed on the aluminum base in the liquid nitrogen with the help of a silver adhesive, and the bases were coated in the gold coating/disperser device (Jouki, Mortazavi, Yazdi, & Koocheki, 2014; Salarbashi et al., 2013).

### 2.6 Statistical analysis

Data of different groups were collected and analyzed using one‐way ANOVA by the SPSS software. In all calculations, *p*‐value of <.05 was considered statistically significant.

## 3 RESULTS

### 3.1 In vitro antimicrobial activity of the active packaging

**Figure 1:**
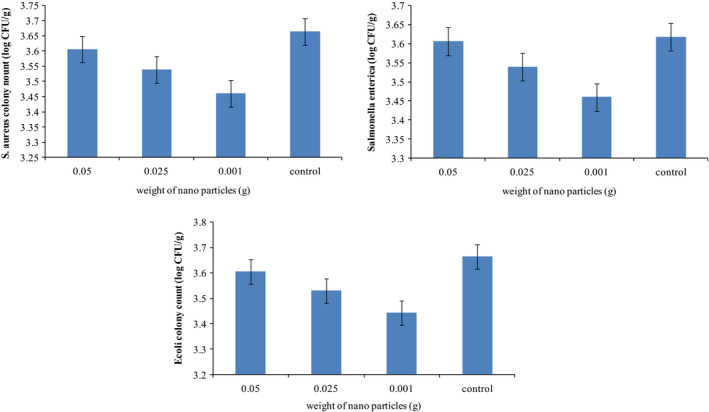
Number of colonies of bacteria (CFU/g) in the presence of new active packaging incorporated with different concentrations of nanoparticles without milk

Antibacterial activity of the new package containing GEO and silica nanoparticles with various concentrations (0.05, 0.025, 0.001 g) of silica nanoparticles against *Staphylococcus aureus*, *Salmonella enterica*, *Escherichia coli*, and *Listeria monocytogenes* is shown in Figure 1. Colony numbers reduced slightly with decrease in the amount of silica nanoparticles, although this reduction was not significant (*p* ≥ .05). In general, GEO with silica nanoparticle incorporated films determined similar antimicrobial activity against all the tested bacteria (*p* ≥ .05). These findings showed that 0.001 g silica nanoparticle has higher inhibitory effect, which is suitable for making an antimicrobial packaging. Therefore, this concentration selected for further experiments. The growths of *Staphylococcus aureus, Salmonella enterica, Escherichia coli*, and *Listeria monocytogenes* in the presence of 0.001 g silica nanoparticles and GEO active packaging in volatile phase without milk are compared.

**Figure 2:**
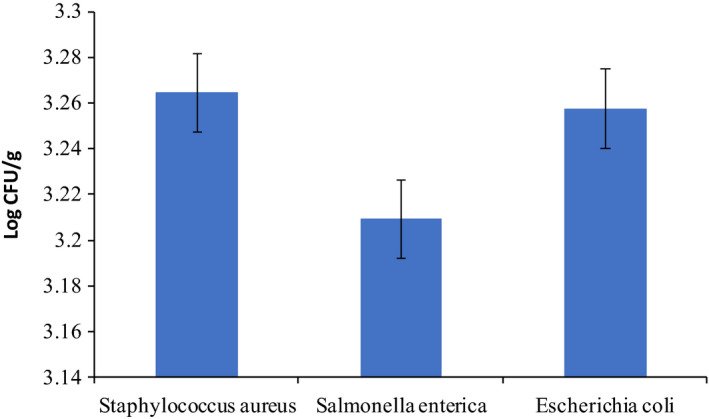
Antibacterial activity (CFU/g) of new active packaging with concentration of 0.001 g silica nanoparticle and GEO without milk during 24 hr

According to Figure 2, GEO has a strong inhibitory effect on *Salmonella*. However, no significant difference was found in the counts of bacteria (*p* ≥ .05).

### 3.2 Antimicrobial activity of the active packaging in milk

**Figure 3:**
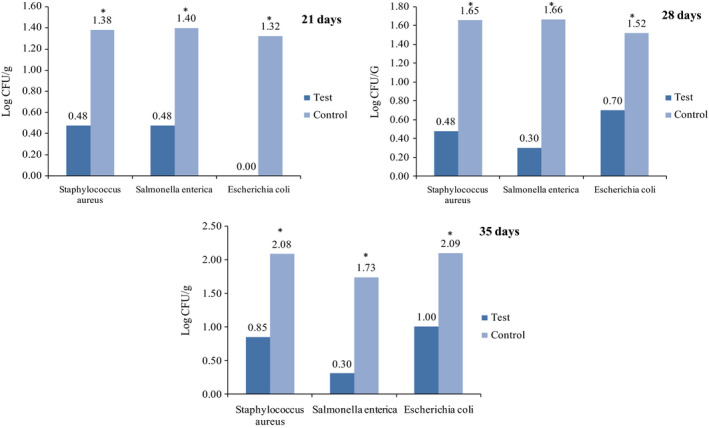
The number of colonies (CFU/g) in the presence of new active packaging and milk within 1–35 days in comparison with the control group. Antibacterial activity of new active packaging with 0.001 g concentration of silica nanoparticle and GEO in milk.(The * showed significant differences)

The colony numbers grown in the presence of milk were evaluated on days 1, 3, 7, 14, 21, 28, and 35 (Figure 3). New milk packaging was able to reduce the growth of all tested bacteria during each storage times (*p* < .05).

### 3.3 Microstructure of the nanomaterial packaging

**Figure 4:**
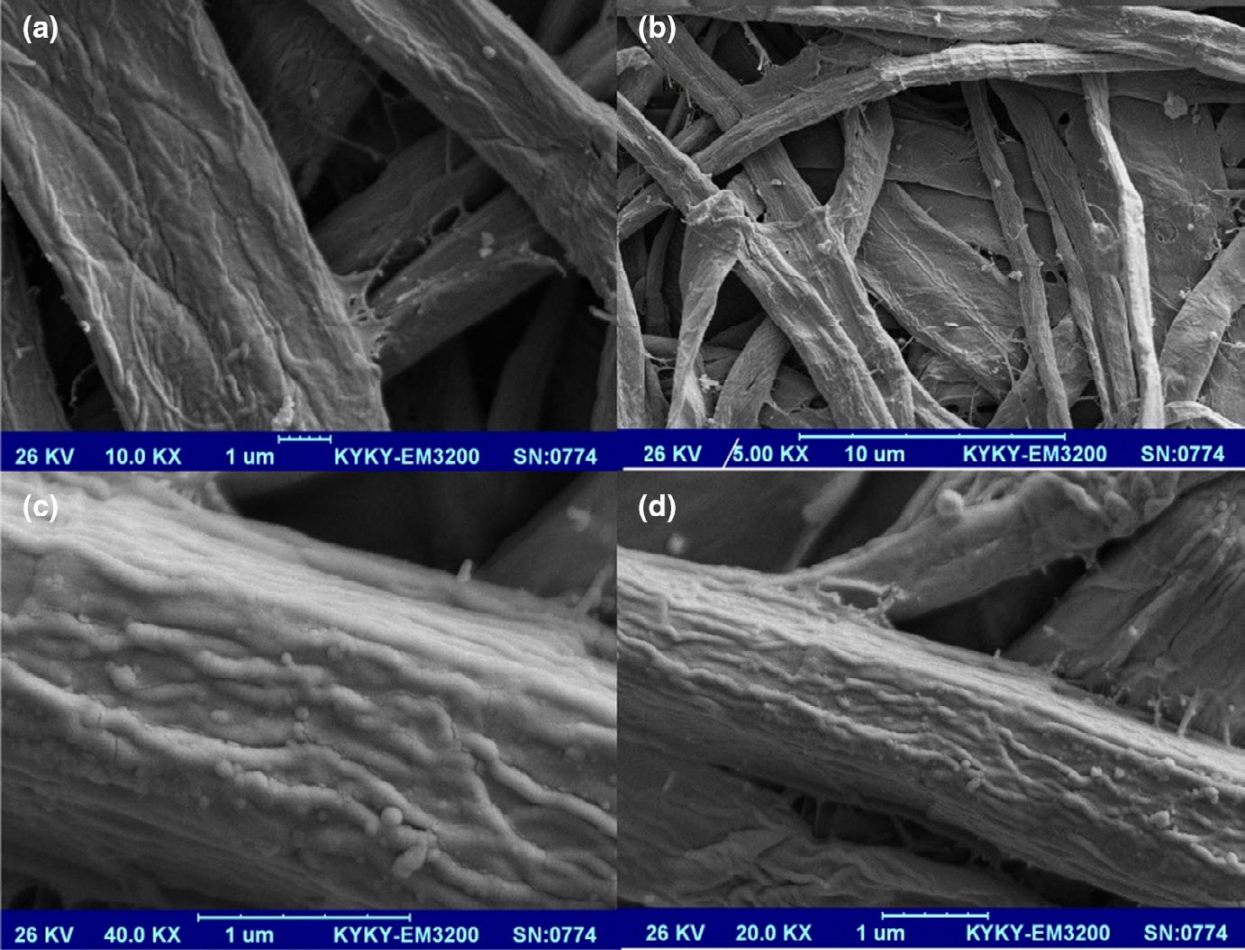
*SEM* micrographs of cellulose without GEO and silica nanoparticles. (Magnitude of pictures (a) 10,000×, (b) 5,000×, (c) 40,000×, (d) 20,000×)

**Figure 5:**
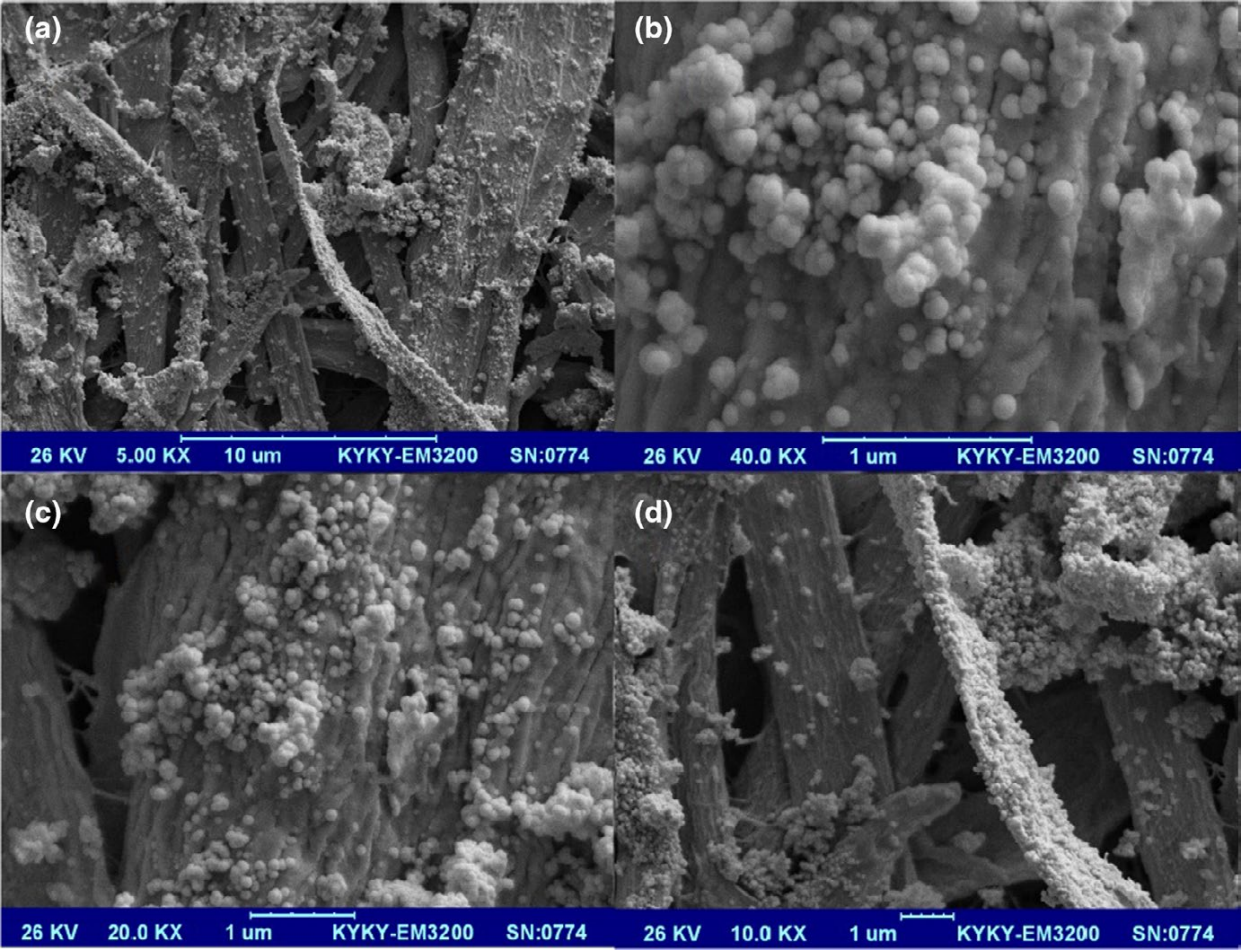
*SEM* micrographs of cellulose with GEO and silica nanoparticles. (Magnitude of pictures (a) 5,000×, (b) 40,000×, (c) 20,000×, (d) 10,000×)

Microstructures of cellulose without and with silica nanoparticles and GEO are shown in Figures 4 and 5, respectively. As shown in Figure 5, the silica nanoparticles and GEO are distributed finely in the smooth and continuous form, which increased the maintenance and stability of GEO.

## 4 DISCUSSION

In recent years, application of new packaging to extend the shelf life of food has been demonstrated by many researchers. Essential oils, classified as Generally Recognized As Safe (GRAS), are one of the antimicrobial agents that extensively used in active packaging (Salarbashi et al., 2013). Incorporating essential oils in packaging is limited due to their volatile nature and organoleptic acceptance. Therefore, applying the essential oils in nano‐scale could solve these problems (Salarbashi et al., 2013). As reported in our previous study, α‐pinene (92.08%) is the main ingredient of *Pistacia atlantica* tree's gum essential oil (Ellahi et al., 2019), which is considered as an antimicrobial agent. Although, other compounds, such as ß‐pinene, sabinene, and camphene, in GEO may contribute to the bacterial inhibitory effect (Akrami et al., 2015; Ellahi et al., 2019).

The inhibitory effect of new packaging containing GEO and 0.001 g silica nanoparticles against the four selected bacteria is shown in Figure 2. Some studies reported that decreasing the silica nanoparticle concentration resulted in higher inhibitory activity due to more release of essential oil. According to our results, the best amount of nanoparticle that had the most antibacterial activity was 0.001 g. The greater inhibitory effect of the active packaging without applying in food system was observed on *S. enterica*.

Akrami et al. investigated the antimicrobial activity of active packaging containing 2%, 4%, and 6% w/w of *Zataria multiflora* essential oil against gram‐positive and gram‐negative bacteria. They concluded that growth of *S. aureus*, *Pseudomonas sp*, *E. coli*, *S. enterica*, and *Listeria innocula* strains was inhibited by using 4% and 6% w/w of *Zataria multiflora* essential oil, while the effective concentration of *Zataria multiflora* essential oil against *S. enterica* and *S. aureus* was 2% (Akrami et al., 2015). Rodriguez et al. used cinnamon volatile oil as an antimicrobial agent at concentrations of 1% to 8% in active packaging. In their study, there was no growth inhibition against *S*. *aureus*, *L. monocytogenes*, and *Enterococcus faecalis*, while the cinnamon volatile oil significantly inhibited the growth of gram‐negative bacteria such as *Salmonella cholerasuis*, *E. coli*, *Yersinia enterocolitica*, and *P. aeruginosa*. The least amount of volatile oil possessing antibacterial activity against gram‐negative bacteria was 4% (Rodriguez et al., 2007).

In recent years, Nanoparticles of clay, chitosan, and starch have been used as active packaging in many studies (Lotfinia, Javanmard Dakheli, & Mohammadi Nafchi, 2018; Pranoto, Rakshit, & Salokhe, 2005; Yahyazadeh, Zare, Omidbaigi, Faghih‐Nasiri, & Abbasi, 2009), while there is no report on the use of silica nanoparticles in active packaging. Yahyazadeh et al. (2009) reported that polyethylene coating containing thyme essential oil and clay nanoparticles had a significant inhibitory effect on the growth of fungus in citrus fruit. In our experiments, using the new packaging resulted in lower bacterial colony count in milk in most storage time intervals, and a significant difference was observed between some groups (*p* < .05). The packaging contained silica nanoparticles without GEO had no antibacterial effects in both vapor phase and the presence of milk. As another control, package incorporated with just GEO in the vapor phase and in the presence of sterile milk showed antibacterial activity only in the first 24 hr. Therefore, the absence of nanoparticles accelerated the release of GEO, so that it would not prolong the shelf life of packed food.

Rodriguez and his colleagues used active packaging containing cinnamon essential oil for preservation of strawberries up to 7 days and achieved longer shelf life of strawberries (Rodriguez et al., 2007). According to another study, the antimicrobial film of plant essential oil increased the shelf life of bread (Lotfinia et al., 2018). Ghasemi, Javadi, Moradi, and Khosravi‐Darani (2015) were able to improve the shelf life of cheese up to 70 days by an active film containing essential oil of *Zataria multiflora*. In our experiment, the antibacterial active packaging used for preserving milk extended the shelf life up to 35 days.

## 5 CONCLUSION

Current study showed that the polypropylene coated with *Pistacia atlantica's* gum EO. Oil and silica nanoparticles have antibacterial properties and can maintain its properties for up to 35 days, both with and without presence of milk. Nanoparticles of silica served to sustain the release of oil. Therefore, this type of packaging can be used for preserving milk. This study may provide insights for extension of further investigations on other foodstuffs.

## CONFLICT OF INTEREST

There is no conflict of interest to declare.
